# MEK Inhibitors and Toll-like Receptor Signaling: Implications for Infection and Inflammation

**DOI:** 10.3390/ijms27135666

**Published:** 2026-06-23

**Authors:** Oliver Planz

**Affiliations:** Institute of Immunology, University and University Hospital of Tübingen, 72076 Tübingen, Germany; oliver.planz@uni-tuebingen.de

**Keywords:** Toll-like receptors, MEK inhibitors, MEK/ERK signaling, innate immunity, infection and inflammation, host-directed therapy, immune modulation

## Abstract

Toll-like receptors (TLRs) are essential components of the innate immune system that enable host cells to sense microbial and endogenous danger signals and to initiate inflammatory and antimicrobial responses. Activation of TLRs triggers complex intracellular signaling networks that culminate in the induction of pro-inflammatory cytokines, type I interferons, and co-stimulatory molecules. In addition to the well-characterized nuclear factor κB (NF-κB) and interferon regulatory factor (IRF) pathways, mitogen-activated protein kinases (MAPKs) play a critical modulatory role in TLR signaling. MAPK/ERK kinase (MEK) inhibitors were originally developed for the treatment of cancer and are widely used in clinical oncology. Accumulating evidence indicates that pharmacological inhibition of MEK/extracellular signal regulated kinase (ERK) signaling profoundly affects immune cell function and TLR-driven responses. Depending on timing, dose, and disease context, MEK inhibition can attenuate excessive inflammation but may also interfere with protective host defense mechanisms. This duality highlights the context-dependent role of MEK/ERK signaling in infection and inflammation. In this review, I summarize current knowledge on the integration of MEK/ERK signaling into TLR-mediated innate immune responses and discuss the immunological consequences of MEK inhibition in infectious and inflammatory settings. By synthesizing mechanistic and translational studies, I aim to provide a framework for understanding MEK inhibitors as immune modulators rather than as broadly acting anti-inflammatory agents.

## 1. Introduction

Toll-like receptors (TLRs) constitute a central component of the innate immune system by enabling host cells to detect conserved pathogen-associated molecular patterns derived from bacteria, viruses, fungi, and parasites. Since their initial characterization, TLRs have been recognized as key sensors that initiate inflammatory and antimicrobial responses at the earliest stages of infection, thereby providing a first line of host defense and shaping subsequent adaptive immune responses [1,2,3]. In humans, ten functional TLRs have been identified, each recognizing distinct microbial ligands and displaying characteristic expression patterns across immune and non-immune cell types [2,4].

Upon ligand engagement, TLRs activate complex intracellular signaling networks that culminate in the induction of pro-inflammatory cytokines, type I interferons, chemokines, and co-stimulatory molecules. These signaling pathways are tightly regulated in order to balance effective pathogen control with the prevention of excessive inflammation and tissue damage [1,2,5,6]. Dysregulated TLR signaling has been implicated in a wide range of pathological conditions, including sepsis, chronic inflammatory diseases, autoimmune disorders, and cancer-associated inflammation, underscoring the importance of precise temporal and spatial control of TLR-driven responses [3,5,7,8,9,10,11].

Canonical TLR signaling is commonly divided into MyD88-dependent and TIR-domain-containing adapter-inducing interferon-β (TRIF)-dependent pathways, both of which converge on transcription factors such as nuclear factor κB (NF-κB), activator protein-1 (AP-1), and interferon regulatory factors (IRFs) [12,13]. While these transcriptional regulators are often viewed as the principal downstream effectors of TLR activation, it has become increasingly clear that mitogen-activated protein kinase (MAPK) pathways play a critical modulatory role by integrating upstream signals and fine-tuning inflammatory outputs [14,15]. MAPKs influence not only the magnitude but also the qualitative nature of cytokine and interferon responses, thereby shaping immune polarization and functional outcomes [14,16].

Among the MAPK pathways activated downstream of TLRs, the MAPK/ERK Kinase (MEK)/ extracellular signal regulated kinase (ERK) signaling axis has emerged as a key regulatory module in innate immune cells. ERK1/2 activation has been shown to control transcriptional and post-transcriptional mechanisms governing cytokine expression, messenger riboxy nuclear acid (mRNA) stability, and feedback inhibition [14,15,17]. Importantly, ERK signaling does not operate as a simple on/off switch but rather as a signal integrator whose duration, amplitude, and cellular context determine distinct biological outcomes [18,19,20]. This characteristic positions MEK/ERK signaling as a central node for the modulation of TLR-driven immune responses.

Mechanistic studies have revealed that ERK activation downstream of several TLRs is mediated by the tumor progession locus 2 (Tpl2), which links receptor engagement to MEK1/2 and ERK1/2 phosphorylation, particularly in macrophages and dendritic cells [21,22]. Genetic ablation or pharmacological inhibition of this pathway has demonstrated its importance for the regulation of both pro-inflammatory cytokines and regulatory mediators such as interleukin-10 (IL-10), as well as for crosstalk with interferon-regulatory signaling pathways [23,24]. These findings highlight the dual role of MEK/ERK signaling in promoting inflammation while simultaneously contributing to negative feedback mechanisms that limit excessive immune activation.

MEK inhibitors were originally developed to suppress aberrant MAPK signaling in cancer, particularly in tumors driven by constitutive activation of rat sarcoma small GTPases (Ras) or rapidly accelerated fibrosarcoma serine/threonine kinases (RAF). Several of these compounds have progressed into clinical use and are now widely applied in oncology [25,26,27,28]. However, early pharmacological studies largely focused on tumor cell–intrinsic effects and paid limited attention to the impact of MEK inhibition on immune signaling pathways. More recent studies have demonstrated that MEK inhibitors exert complex and context-dependent immunomodulatory effects, influencing T-cell activation and differentiation, dendritic cell function, and innate immune signaling pathways within inflammatory and tumor microenvironments [29,30,31].

In the context of infection and inflammation, these immunomodulatory properties are of relevance. On the one hand, pharmacological MEK inhibition can attenuate excessive inflammatory responses and limit immune-mediated tissue injury. On the other hand, MEK/ERK signaling contributes to effective antimicrobial defense mechanisms, raising concerns that inappropriate pathway inhibition may alter host protection against infection [14,15,32]. This apparent dichotomy underscores the context-dependent nature of MEK/ERK signaling in innate immunity and highlights the need for a nuanced understanding of its role downstream of TLRs.

In this review, I summarize current knowledge on the role of MEK/ERK signaling in TLR-mediated innate immune responses, with a particular focus on the immunological consequences of pharmacological MEK inhibition. I discuss how MEK/ERK integrates into canonical TLR signaling pathways, how MEK inhibitors modulate cytokine and interferon responses, and how these effects translate into outcomes in infection and inflammation. Unlike previous reviews focusing primarily on MAPK signaling or the oncological application of MEK inhibitors, this review specifically integrates current knowledge on the role of MEK/ERK signaling in TLR-driven innate immune responses and discusses the immunological consequences of MEK inhibition across infection, sterile inflammation, and autoimmunity. By synthesizing data from molecular, cellular, and translational studies, this review aims to provide a framework for understanding MEK inhibitors as context-dependent modulators of innate immunity rather than as purely anti-inflammatory agents.

## 2. TLR Signaling Pathways with a Focus on MEK/ERK

TLR signaling is initiated upon recognition of microbial or endogenous ligands at the cell surface or within endosomal compartments, leading to the recruitment of adaptor proteins and the activation of downstream kinase cascades. Canonically, TLR signaling is divided into MyD88-dependent and TRIF-dependent pathways, which differ in adaptor usage, subcellular localization, and transcriptional outputs [33,34]. Despite these differences, both pathways converge on a limited number of signaling modules that coordinate inflammatory and antiviral responses [35,36].

The MyD88-dependent pathway is engaged by most TLRs and leads to the rapid activation of NF-κB and MAPKs through the sequential recruitment of IRAK kinases and TRAF6, culminating in transforming growth factor-β-activated kinase 1 (TAK1) activation [37,38,39]. In contrast, TRIF-dependent signaling, most prominently associated with TLR3 and TLR4, preferentially activates IRF3 and IRF7 via TANK-binding kinase 1 (TBK1) and IκB kinase ε (IKKε), resulting in the induction of type I interferons [10,13]. Importantly, both signaling routes also activate MAPK cascades, underscoring their central role in integrating diverse TLR-derived signals.

Mitogen-activated protein kinases constitute a family of serine/threonine kinases that include ERK1/2, p38, and c-Jun N-terminal kinase (JNK), each of which contributes distinct regulatory functions to innate immune signaling [14,40,41]. While p38 and JNK are often associated with stress responses and pro-inflammatory gene expression, ERK1/2 signaling has emerged as a more nuanced regulator of inflammatory outcomes [19,41,42]. ERK activation influences transcriptional activity, mRNA stability, translational efficiency, and post-translational modifications, thereby shaping both the magnitude and quality of TLR-induced responses [14,19,43].

Among the MAPK pathways activated downstream of TLRs, the MEK/ERK axis is unique in its strong dependence on upstream scaffold and adaptor proteins that confer cell type–specific signaling characteristics. In myeloid cells, ERK activation downstream of TLRs is predominantly mediated by the Tpl2, which couples receptor engagement to MEK1/2 and ERK1/2 phosphorylation [21]. Tpl2 is held in an inactive complex with NF-κB1 p105 under resting conditions and is released upon IκB kinase (IKK)-mediated phosphorylation and degradation of p105 following TLR stimulation [44]. This mechanism directly links NF-κB activation to ERK signaling and provides a molecular basis for coordinated inflammatory regulation.

Genetic studies have demonstrated that Tpl2 deficiency selectively impairs ERK activation downstream of several TLRs without globally disrupting NF-κB signaling, highlighting the specificity of this pathway [21,45,46]. Functionally, loss of Tpl2 or pharmacological inhibition of MEK/ERK signaling results in altered cytokine production profiles, including reduced expression of certain pro-inflammatory mediators and dysregulated induction of regulatory cytokines such as IL-10 [24,47,48]. These findings underscore the role of MEK/ERK signaling not as a simple amplifier of inflammation but as a critical modulator of cytokine balance.

ERK signaling also intersects with interferon-regulatory pathways, particularly in the context of TRIF-dependent TLR activation. Studies in macrophages and dendritic cells have shown that Tpl2–MEK–ERK signaling negatively regulates type I interferon production by limiting IRF3 activation and interferon-β transcription [23]. This negative regulatory function suggests that ERK signaling contributes to feedback mechanisms that prevent excessive or prolonged interferon responses, which can otherwise lead to immunopathology. Thus, MEK/ERK signaling plays a dual role by promoting inflammatory gene expression while simultaneously constraining antiviral signaling pathways.

In addition to its role in cytokine regulation, MEK/ERK signaling influences cellular differentiation and activation states within the innate immune system. ERK activity has been implicated in dendritic cell maturation, antigen processing, and the expression of co-stimulatory molecules required for effective T cell priming [49,50]. The strength and duration of ERK activation appear to be particularly important in determining whether dendritic cells adopt immunogenic or tolerogenic phenotypes, further emphasizing the context-dependent nature of this pathway.

Importantly, MAPKs, including ERK, are integral components of negative feedback circuits that limit TLR signaling. Induction of MAPK phosphatases and other inhibitory molecules serves to terminate signaling and restore cellular homeostasis [51]. Pharmacological inhibition of MEK/ERK signaling therefore has the potential to disrupt these regulatory loops, leading to unintended consequences for immune regulation. This consideration is particularly relevant when interpreting experimental studies employing MEK inhibitors, as observed effects may reflect both direct suppression of inflammatory signaling and interference with endogenous feedback mechanisms.

**Figure 1:**
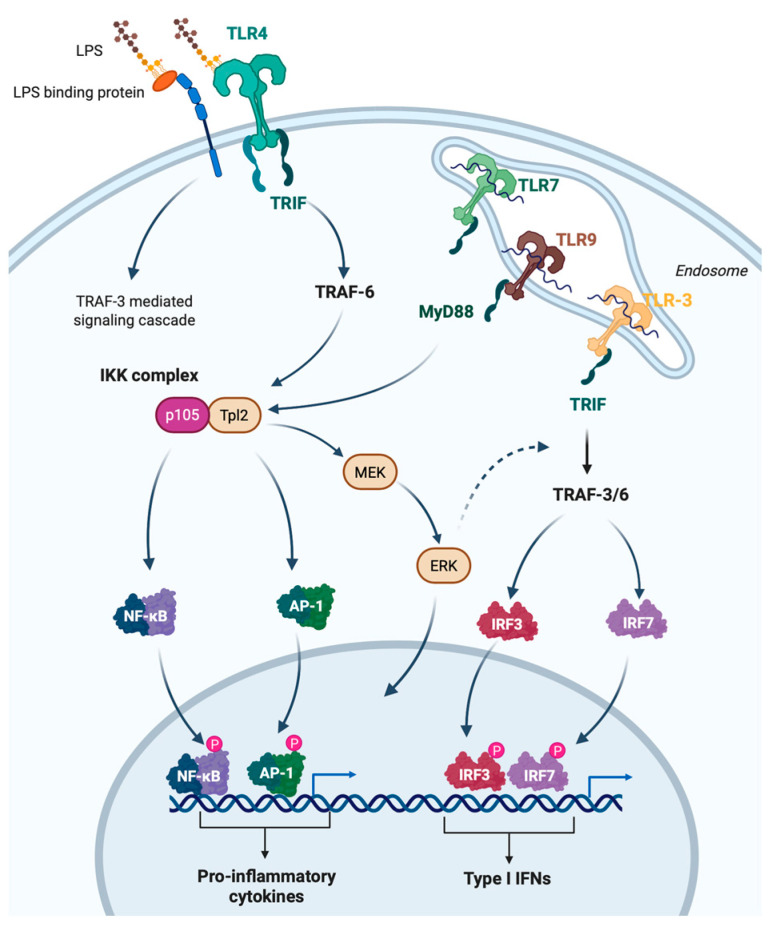
MEK/ERK signaling as an integrative node in Toll-like receptor–mediated innate immune responses. TLRs located at the cell surface or in endosomal compartments activate MyD88- and/or TRIF-dependent signaling pathways upon ligand engagement. In addition to canonical NF-κB and IRF activation, TLR signaling converges on the Tpl2–MEK–ERK axis, which functions as a central integrative module. MEK/ERK signaling modulates inflammatory cytokine production, regulatory feedback mechanisms, and antiviral type I interferon responses in a context- and cell type–dependent manner. Pharmacological inhibition of MEK interferes with this signaling hub, thereby altering the balance between inflammation, immune regulation, and host defense. The figure was created in BioRender. Template adapted from: Natalya Odoardi Clinical Research Coordinator, Children’s Hospital of London.

Taken together, current evidence supports a model in which MEK/ERK signaling functions as an integrative node within TLR signaling networks, linking receptor engagement to finely tuned inflammatory and antiviral outcomes. Rather than acting as a linear downstream effector, the MEK/ERK pathway modulates signal integration, feedback regulation, and cell type–specific responses (Figure 1). This central positioning provides a mechanistic rationale for the profound immunomodulatory effects observed upon pharmacological MEK inhibition and sets the stage for understanding how MEK inhibitors influence infection and inflammation in vivo.

## 3. Pharmacology of MEK Inhibitors Relevant to Immune Modulation

MEK enzymes MEK1 and MEK2 occupy a central position within the canonical RAF–MEK–ERK signaling cascade and serve as the exclusive upstream activators of ERK1/2. Pharmacological inhibition of MEK was originally pursued as a strategy to suppress aberrant MAPK signaling in cancer, particularly in tumors driven by constitutive RAS or RAF activation. As a result, MEK inhibitors represent one of the best-characterized classes of targeted kinase inhibitors in clinical use [25,52,53,54]. However, the same pathway that drives malignant proliferation also plays fundamental roles in immune cell signaling, raising important questions regarding the immunological consequences of MEK inhibition. A structured overview of pharmacological inhibitors targeting different nodes of the Tpl2–MEK–ERK axis is provided in Table 1.

Early MEK inhibitors such as PD98059 and U0126 were developed as research tools and provided initial proof-of-concept that selective blockade of MEK effectively suppresses ERK activation and downstream transcriptional responses [76]. Although these compounds lack the pharmacokinetic properties required for clinical application, they were instrumental in defining the role of MEK/ERK signaling in immune cells, including macrophages and dendritic cells. Subsequent generations of MEK inhibitors, including selumetinib, trametinib, cobimetinib, and binimetinib, exhibit improved potency, selectivity, and bioavailability, enabling their widespread use in both preclinical and clinical settings (Table 2) [25,54,77].

From a pharmacological perspective, MEK inhibitors are generally classified as allosteric inhibitors that bind to MEK in a manner distinct from ATP-competitive kinase inhibitors. This binding mode confers a high degree of selectivity for MEK1/2 and limits off-target effects on other kinases [78,79]. Nevertheless, because MEK/ERK signaling operates as a tightly regulated signaling hub, pharmacological inhibition can have broad downstream consequences that extend beyond the immediate suppression of ERK phosphorylation. In immune cells, these effects are particularly pronounced due to the role of ERK in integrating signals from pattern recognition receptors, cytokine receptors, and costimulatory molecules.

A critical feature of MEK/ERK signaling relevant to immune modulation is its sensitivity to signal strength, duration, and timing. Unlike pathways that function as binary switches, ERK signaling encodes information through graded and temporally controlled activation patterns. Pharmacological MEK inhibition therefore does not merely “turn off” signaling but reshapes the signaling landscape by altering activation thresholds and feedback regulation. This property is especially relevant in innate immune cells, where ERK signaling contributes to both inflammatory activation and negative feedback mechanisms downstream of TLRs [18,80,81,82].

Several studies have demonstrated that MEK inhibitors profoundly influence cytokine production profiles in innate immune cells. In macrophages and dendritic cells, MEK inhibition can suppress the production of pro-inflammatory mediators such as tumor necrosis factor (TNF), IL-6, and IL-1β, while simultaneously affecting regulatory cytokines including IL-10 [14,22,83,84]. Importantly, these effects are context-dependent and vary with stimulus, cell type, and inhibitor concentration. Such findings underscore that MEK inhibitors act as immunomodulators rather than as broadly immunosuppressive agents.

In addition to cytokine regulation, MEK inhibitors affect antigen presentation, dendritic cell maturation, and costimulatory molecule expression. Pharmacological blockade of MEK/ERK signaling has been shown to alter the expression of MHC molecules and co-stimulatory receptors, thereby influencing the capacity of dendritic cells to prime T cell responses. These effects are consistent with the role of ERK signaling in controlling activation thresholds and differentiation states within the innate immune system, as discussed in the preceding section [85,86,87,88].

Another important aspect of MEK inhibitor pharmacology is their impact on feedback regulation within signaling networks. MEK/ERK signaling induces multiple negative feedback loops, including the expression of MAPK phosphatases and inhibitory adaptor proteins, which serve to terminate signaling and restore cellular homeostasis [89,90,91]. Pharmacological inhibition of MEK disrupts these feedback circuits and may thereby produce non-linear or paradoxical effects, particularly during prolonged exposure [92,93]. This consideration is critical for interpreting experimental studies and for understanding the divergent immunological outcomes observed in different disease models.

The immunomodulatory effects of MEK inhibitors have also been explored in the context of infection and inflammation. In viral infection models, MEK inhibition has been shown to suppress viral replication by targeting host cell signaling pathways required for efficient virus propagation, while simultaneously modulating innate immune responses [59,60,61,94,95,96]. In inflammatory settings, MEK inhibitors can attenuate excessive cytokine production and tissue damage but may also compromise protective immune responses if administered inappropriately [86,97]. These observations highlight the narrow therapeutic window within which MEK inhibition may confer benefit without inducing deleterious immunosuppression.

Taken together, the pharmacological properties of MEK inhibitors position them as powerful tools for modulating immune signaling pathways. Their high selectivity for MEK1/2, combined with the central role of MEK/ERK signaling in innate immunity, provides a mechanistic basis for their profound and context-dependent immunological effects. Understanding how pharmacological parameters such as dose, timing, and duration of MEK inhibition intersect with immune signaling dynamics is essential for the rational application of these compounds in infection and inflammation.

## 4. Effects of MEK Inhibition on TLR-Driven Innate Immune Responses

TLR activation induces complex innate immune responses that are tightly regulated in magnitude and duration to ensure effective host defense while limiting immunopathology. In addition to canonical transcriptional pathways mediated by NF-κB and IRFs, MEK/ERK signaling plays a central role in shaping TLR-driven outputs by modulating cytokine production, interferon responses, and feedback regulation. Pharmacological inhibition of MEK therefore exerts profound effects on innate immune responses initiated by TLR engagement.

A substantial body of evidence demonstrates that MEK/ERK signaling contributes to the regulation of pro-inflammatory cytokines downstream of TLR activation. In macrophages stimulated with TLR ligands such as lipopolysaccharide (TLR4) or lipopeptides (TLR2), MEK inhibition suppresses the production of TNF-α, IL-6, and IL-1β, reflecting the involvement of ERK in transcriptional and post-transcriptional control mechanisms. These effects are mediated in part by ERK-dependent regulation of transcription factor activity and mRNA stability, highlighting the role of MEK/ERK signaling in fine-tuning inflammatory outputs.

Importantly, the impact of MEK inhibition on cytokine production is highly context dependent. Differences in cell type, stimulus, inhibitor concentration, and timing of intervention can result in divergent outcomes, ranging from marked attenuation of inflammation to relatively subtle modulatory effects [97,98,99,100]. These observations underscore that MEK inhibitors do not function as uniform anti-inflammatory agents but instead reshape inflammatory responses in a stimulus-specific manner.

Beyond pro-inflammatory mediators, MEK/ERK signaling plays a key role in the induction of regulatory cytokines, particularly IL-10. ERK activation downstream of TLRs promotes IL-10 production in macrophages and dendritic cells, thereby contributing to negative feedback regulation and resolution of inflammation [23,24]. Pharmacological MEK inhibition disrupts this regulatory axis and can lead to reduced IL-10 expression, potentially shifting the balance toward heightened or prolonged inflammatory responses. This dual role of MEK/ERK signaling—supporting both inflammatory and regulatory pathways—highlights its function as an integrative signaling node rather than a simple pro-inflammatory pathway. The net immunological outcome of MEK inhibition therefore depends on the relative contribution of ERK to pro- versus anti-inflammatory programs under specific conditions of TLR activation [23,101,102]. In addition to cytokine and interferon regulation, MEK/ERK signaling also contributes to innate antimicrobial defense mechanisms through the modulation of antimicrobial peptide expression downstream of TLR activation. Antimicrobial peptides such as defensins and cathelicidins represent important effector molecules of the innate immune response by directly limiting microbial growth and shaping local immune environments. Experimental studies have demonstrated that pharmacological inhibition of MEK/ERK signaling can alter TLR-induced antimicrobial peptide expression in epithelial and immune cells, thereby influencing host defense functions beyond classical cytokine regulation. These findings further emphasize that MEK inhibition may affect not only inflammatory signaling pathways but also direct antimicrobial effector mechanisms in a context-dependent manner [103,104]. This aspect may be particularly relevant at barrier tissues such as the respiratory tract and skin, where TLR-driven antimicrobial peptide production contributes substantially to early pathogen control.

In this context, MEK inhibition can lead to enhanced or prolonged type I interferon responses, reflecting the release of ERK-mediated inhibitory constraints. These findings are particularly relevant in viral infections, where type I interferons play a critical role in antiviral defense but may also contribute to immunopathology if excessively produced. Thus, MEK inhibition has the potential to alter the balance between antiviral efficacy and inflammatory damage by modulating interferon signaling downstream of TLRs.

TLR-driven activation of dendritic cells is essential for the initiation of adaptive immune responses. MEK/ERK signaling influences dendritic cell maturation, antigen processing, and the expression of co-stimulatory molecules required for effective T cell priming. Pharmacological inhibition of MEK has been shown to alter the phenotypic and functional maturation of dendritic cells following TLR stimulation, resulting in changes in antigen presentation capacity and T cell activation [49,84,105]. These effects further emphasize the role of MEK/ERK signaling in controlling activation thresholds and functional differentiation states within the innate immune system. By modulating dendritic cell responses to TLR ligands, MEK inhibitors indirectly shape downstream adaptive immune responses, with potential implications for vaccination, chronic infection, and immune tolerance.

An important but often underappreciated aspect of TLR signaling is the presence of intrinsic feedback mechanisms that limit signal duration and prevent excessive inflammation. MAPK pathways, including ERK, induce the expression of dual-specificity phosphatases and other inhibitory molecules that serve to terminate signaling and restore cellular homeostasis [89,90]. MEK inhibition disrupts these feedback loops, which may lead to non-linear signaling behavior and compensatory activation of parallel pathways. Such disruption of feedback regulation may contribute to the diverse and sometimes paradoxical effects observed following MEK inhibition in innate immune cells. These considerations are particularly relevant for prolonged or systemic MEK inhibition, where cumulative effects on signaling networks may influence immune homeostasis.

Collectively, the effects of MEK inhibition on TLR-driven innate immune responses are highly context-dependent. In settings characterized by excessive inflammation, MEK inhibition may confer therapeutic benefit by dampening cytokine production and limiting tissue damage. Conversely, in situations where robust innate immune activation is required for pathogen control, MEK inhibition may compromise host defense mechanisms.

This duality highlights the need for careful consideration of disease context, timing, and dosage when targeting MEK/ERK signaling in infection and inflammation. Rather than functioning as broadly immunosuppressive agents, MEK inhibitors should be viewed as modulators of innate immune signaling whose effects depend on the underlying immunological landscape.

## 5. MEK Inhibitors in Infection Models

Host-directed inhibition of the RAF–MEK–ERK pathway has emerged as a broadly applicable antiviral strategy because many clinically relevant RNA viruses exploit ERK signaling to support efficient replication, assembly, or egress [106,107,108,109,110,111]. In contrast to direct-acting antivirals (DAAs), MEK inhibitors impose a higher barrier to resistance development and can additionally modulate infection-associated hyperinflammation—an important consideration in severe respiratory viral disease. Recent work has strengthened the concept that MEK inhibition can confer a dual benefit through (i) antiviral restriction and (ii) immunomodulation that mitigates pathogenic inflammation, depending on timing, dosing, and disease stage [111].

Influenza A virus (IAV) is one of the best-characterized examples of MEK/ERK dependency among respiratory viruses. In preclinical models, host-targeted MEK inhibition reduces viral propagation and can attenuate infection-associated cytokine expression. A clinically approved MEK inhibitor (trametinib) was shown to efficiently block IAV replication across subtypes in vitro, and to dampen virus-induced cytokine expression, supporting the feasibility of repurposing clinically used MEK inhibitors for acute influenza [112]. Zapnometinib (ATR-002), a non-ATP-competitive MEK1/2 inhibitor developed for acute viral infections, has been studied extensively in influenza models. In a comparative preclinical analysis, zapnometinib, the active metabolite of CI-1040 displayed favorable pharmacokinetic properties and robust in vivo efficacy in reducing influenza viral load in mice despite lower apparent potency in cell culture, illustrating how tissue exposure and target engagement can dominate antiviral outcomes in vivo [64]. Importantly, recent immunology-focused work suggests that MEK inhibition may also shape disease course by modifying immunoregulatory circuits: zapnometinib treatment reduced the induction of regulatory T cells (Tregs) after influenza A virus infection and improved disease progression in a mouse model, consistent with the notion that MEK/ERK activity contributes to immune programs that—while regulatory—can be maladaptive in certain settings of severe influenza [68]. These influenza studies collectively support a mechanistic framework in which MEK inhibition suppresses virus-permissive host signaling while rebalancing inflammatory and regulatory pathways. At the same time, they underscore the need to define “therapeutic windows” where antiviral and immunomodulatory effects align (e.g., early replication phase vs. later hyperinflammatory phase), as the immune consequences of MEK inhibition may differ substantially depending on disease stage [111].

The COVID-19 pandemic accelerated clinical translation of host-directed strategies aimed at late-stage disease where hyperinflammation dominates and DAAs may be less effective. Zapnometinib has advanced into clinical evaluation and provided proof-of-concept in hospitalized COVID-19 patients. In the RESPIRE randomized, placebo-controlled Phase 2 trial, zapnometinib demonstrated an acceptable safety profile and showed trends toward clinical improvement in prespecified subgroups, supporting feasibility of RAF–MEK–ERK targeting in moderate/severe viral respiratory infection [69]. Beyond clinical outcomes, more recent translational analyses emphasize the immunomodulatory component of MEK inhibition in COVID-19. In a 2025 study integrating animal models and patient samples, zapnometinib was associated with antiviral activity and “favorable” immunomodulatory effects, reinforcing the dual-benefit concept in coronavirus-driven lung disease [65]. In parallel, dedicated pharmacokinetics (PK)/ pharmacodynamics (PD) work in animals and healthy human volunteers has provided target engagement and exposure data for zapnometinib, which is essential for rationally linking dosing to both antiviral efficacy and immune modulation across species [67].

A major rationale for host-targeted antivirals is the reduced likelihood of selecting resistant viral variants. In the influenza context, this principle is supported by the host-dependency of ERK pathway activation for efficient replication and by preclinical data showing robust antiviral effects without targeting viral enzymes directly [64]. For coronaviruses, host-directed MEK inhibition has also been investigated as a partner for DAAs, aiming to combine a high resistance barrier (host target) with potent virus-directed inhibition [70]. This combination logic is conceptually attractive for severe disease where viral replication and dysregulated host responses can overlap temporally, although optimal schedules remain to be defined.

While many infection studies focus on antiviral outcomes, immune modulation by MEK inhibitors can also produce unfavorable effects under certain inflammatory conditions. A recent report demonstrated that MEK inhibitor treatment increased mortality in a murine lipopolysaccharide (LPS)-induced inflammation model via an IL-12–nitric oxide (NO) axis, a finding that highlights the possibility of stimulus-specific and context-dependent adverse immune phenotypes when MEK/ERK signaling is pharmacologically perturbed [113]. These results are an important counterpoint to antiviral benefit studies and support the need for careful patient stratification, dosing, and monitoring—particularly in bacterial sepsis-like syndromes or endotoxemia-dominant inflammatory states.

Collectively, infection model data support MEK inhibitors as host-directed agents with a potentially unique therapeutic profile: suppression of virus-permissive host signaling and modulation of inflammatory trajectories in hyperinflammatory respiratory viral disease. Clinical translation is now supported by randomized trial evidence in COVID-19 and expanding mechanistic datasets across respiratory viruses [69]. However, the same central pathway role that enables broad antiviral activity also implies a narrow context-dependent safety window, as illustrated by exacerbation of endotoxin-driven lethality in preclinical models [113]. Future work should therefore prioritize (i) biomarkers of pathway engagement in relevant immune compartments, (ii) dosing schedules aligned with disease stage (replicative vs. hyperinflammatory), and (iii) combination regimens that preserve protective host defense while limiting immunopathology.

## 6. Mek Inhibition in Sterile Inflammation and Autoimmunity

Sterile inflammation is initiated by endogenous danger signals (DAMPs) released during cell stress, necrosis, tissue remodeling, or ischemia–reperfusion, and is increasingly understood as a pattern recognition receptor (PRR)-driven process rather than a “non-specific” inflammatory reaction [114,115,116,117,118]. Among PRRs, TLR2 and TLR4 are particularly relevant in sterile settings because they can be triggered by diverse host-derived ligands and, together with co-receptors and accessory molecules, translate tissue damage into MyD88- and/or TRIF-dependent transcriptional programs. Recent syntheses emphasize that DAMP identity, compartmentalization, and receptor context determine the qualitative inflammatory outcome and the transition toward resolution—features that are central to chronic sterile inflammatory diseases and autoimmunity. In this framework, MEK/ERK signaling is best viewed as a tunable amplifier and “decision layer” downstream (and in feedback around) TLR pathways, shaping both the magnitude and the character of inflammatory outputs [119,120].

In sterile tissue injury, TLR engagement drives cytokine production, leukocyte recruitment, and local stromal activation—processes that often become maladaptive when DAMP exposure is persistent. Mechanistically, TLR2/4 signaling engages MAPK modules (including ERK) alongside NF-κB and IRFs, enabling parallel control of transcription, mRNA stability, and translation of inflammatory mediators. Because ERK activity can integrate inputs from TLRs, cytokine receptors, and growth factor pathways present in inflamed tissues, it is positioned to govern “inflammatory tone” under conditions where multiple cues coexist (e.g., hypoxia, oxidized lipids, extracellular matrix fragments). This integrative role is especially pertinent to sterile inflammation where no pathogen-derived “off switch” exists and resolution depends on the balance of feedforward and negative-feedback circuits [119,120].

Across multiple experimental systems, MEK inhibitors suppress hallmark TLR-induced cytokines (notably TNF and IL-1 family outputs) and can reduce systemic inflammatory injury [121]. For example, trametinib blocks MEK–ERK activation downstream of TLR4 stimulation and suppresses TNF production in macrophages and PBMCs, protecting mice from LPS-induced endotoxin shock—an archetypal TLR4-driven cytokine storm model (while not “sterile,” it cleanly illustrates MEK/ERK’s leverage over TLR outputs) [122]. In a clinically oriented polymicrobial sepsis model, delayed trametinib treatment attenuated systemic cytokine elevations and multi-organ injury, consistent with MEK/ERK acting as a modifiable node within PRR/TLR-triggered inflammatory cascades [123]. More recently, MEK1/2 inhibitors (including PD0325901, trametinib, CI-1040) were shown to reduce TLR2- and TLR4-dependent pro-inflammatory responses in human macrophages from people with cystic fibrosis, without broadly crippling phagocyte antimicrobial functions—supporting the concept that MEK inhibition may “de-intensify” harmful inflammation while preserving core innate effector capacity in some settings [97]. However, autoimmunity and sterile inflammation also highlight an important caveat: MEK/ERK can exert negative control over specific antiviral-like programs, particularly type I interferon (IFN) in nucleic-acid–sensing contexts. In plasmacytoid dendritic cells, MEK1/2 inhibition (PD0325901, U0126) potentiated TLR7/9-driven type I IFN production in a human plasmacytoid dendritic cell (pDC) model, underscoring that MEK blockade may increase selected inflammatory outputs depending on cell type and pathway wiring [124]. Thus, in sterile/autoimmune disease—where endosomal TLRs (TLR7/8/9) and IFN signatures can be pathogenic—MEK inhibition could be beneficial or detrimental depending on whether the dominant disease axis is cytokine/TNF/IL-1–skewed versus IFN-driven.

In rheumatoid arthritis (RA), TLR2/4 stimulation by endogenous ligands in synovial tissue is widely implicated in sustaining macrophage and fibroblast-like synoviocyte activation, creating a cytokine-rich environment that perpetuates sterile inflammation. In vivo, the MEK inhibitor PD184352 reduced disease in collagen-induced arthritis and mechanistic analyses supported a central pro-inflammatory role for MEK/ERK signaling within the arthritic joint environment [125]. Complementing this, combinatorial pathway work in collagen-induced arthritis suggested that targeting ERK/MEK alongside mTOR signaling can yield additive benefit, consistent with ERK functioning as part of a broader activation/translation network in pathogenic T cells and myeloid cells in chronic inflammation [126]. Importantly, negative-feedback regulation of ERK is also disease-relevant: deletion of the ERK-directed phosphatase dual-specificity phosphatase 6 (DUSP6) protected mice and reduced severity in autoimmune arthritis, highlighting that the amplitude and duration of ERK activity—regulated by phosphatases induced during innate activation—can materially shape chronic sterile inflammation [127].

Psoriasis provides a useful TLR-linked sterile inflammation model because topical imiquimod activates TLR7/8 and triggers a robust IL-23/IL-17–dominated inflammatory dermatitis in mice [128,129]. While many mechanistic intervention studies use ERK inhibitors rather than clinically approved MEK inhibitors, the pathway-level inference is informative: ERK inhibition (JSI287) alleviated IMQ-induced skin pathology and reduced inflammatory cytokine signatures, consistent with MEK/ERK supporting downstream amplification of TLR7/8-initiated networks that promote Th17-associated inflammation [128]. This aligns with the broader idea that MEK/ERK activity can reinforce chronic inflammatory circuits in barrier tissues where innate sensing and adaptive polarization are tightly coupled.

Finally, in autoimmune conditions characterized by endosomal TLR engagement and type I IFN signatures (e.g., lupus-like settings), MEK/ERK biology may be particularly nuanced. Because MEK/ERK can restrain (and MEK inhibition can enhance) TLR7/9-driven IFN-I in pDC contexts, careful alignment of therapeutic intent (e.g., suppressing TNF/IL-1 vs. suppressing IFN pathways) is essential when considering MEK inhibitors for sterile inflammatory or autoimmune indications [124].

A practical way to re-integrate TLRs into the sterile inflammation/autoimmunity discussion is to frame MEK/ERK as part of a feedback-governed signaling economy. Sterile inflammation requires not only initiation via DAMP-sensing (often via TLR2/4) but also an orderly transition toward resolution [130,131]. Reviews on sterile inflammation emphasize receptor- and mediator-defined macrophage state transitions as a critical determinant of chronicity [120]. Within this, dual-specificity phosphatases (DUSPs) and related feedback regulators shape the time course of MAPK activation in innate cells, thereby influencing whether TLR signals remain self-limited or become persistent [90]. Disease and genetics models (e.g., DUSP6 in arthritis) reinforce that altering ERK feedback control can shift outcomes substantially, supporting the view that therapeutic MEK inhibition will interact with pre-existing feedback “set points” in inflamed tissues [127]. Accordingly, MEK inhibitors may be most attractive where pathology is driven by sustained TLR2/4→cytokine circuits and maladaptive amplification, whereas IFN-dominant endosomal TLR states may warrant a more cautious, biomarker-guided approach [124].

## 7. Therapeutic Implications and Risks

MEK inhibitors are increasingly recognized as context-dependent modulators of innate immunity rather than uniform anti-inflammatory agents [14,132]. Because the RAF–MEK–ERK axis sits at a convergence point of TLR signaling, cytokine receptor signaling, and stress/growth-factor inputs, MEK inhibition can simultaneously reshape (i) pro-inflammatory cytokine programs, (ii) regulatory feedback responses, and (iii) antiviral interferon pathways. This central network position provides a mechanistic rationale for the broad phenotypes observed in experimental models and in early clinical translation, but it also implies that therapeutic benefit is likely to depend strongly on timing, dose, duration, and the dominant inflammatory endotype of a given disease [5,42,77,132,133,134].

Hyperinflammatory respiratory viral disease represents a major opportunity for host-directed MEK inhibition, because ERK signaling can be required for efficient replication of several respiratory viruses while also contributing to inflammatory pathology. Conceptually, MEK inhibitors may provide a “dual-benefit” profile by (i) restricting virus-permissive host signaling and (ii) reducing damaging inflammation, especially when administered early enough to influence both replication and the inflammatory trajectory. This rationale is supported by contemporary reviews emphasizing MEK inhibitors as host-targeted antivirals in hyperinflammatory respiratory viral diseases [111,135,136].

Clinical translation has advanced most clearly for zapnometinib (ATR-002), which was evaluated in hospitalized COVID-19 patients in the RESPIRE randomized phase 2 trial, providing a controlled clinical dataset on feasibility and safety in a severe viral respiratory setting [69]. While broader efficacy conclusions require larger studies, the existence of randomized clinical evidence is a key milestone for host-directed MEK inhibition in infection. In influenza, U0126, CI-1040, zapnometinib and the clinically approved MEK inhibitor trametinib demonstrated strong antiviral suppression and reduction of cytokine expression in preclinical models, supporting feasibility of MEK inhibition as a host-directed antiviral approach in principle [58,60,64,96,112].

Beyond infection, MEK inhibition may be beneficial in selected TLR-driven sterile inflammatory states (e.g., excessive TLR2/4 cytokine networks), where ERK contributes to inflammatory amplification. In such settings, MEK inhibitors could be positioned as targeted dampeners of pathologic innate activation, potentially complementing established cytokine-directed therapies [14,137].

A recurring translational lesson is that MEK inhibition does not simply suppress inflammation; instead it reprograms innate signaling trajectories. ERK signaling encodes information through amplitude and duration, and it is embedded in feedback circuits that govern tolerance, priming, and resolution. Prolonged inhibition of the MEK1/2–ERK axis can yield non-linear outcomes, including reversal of tolerance and priming toward enhanced expression of IL-1β and other inflammatory mediators in macrophages after extended blockade—an observation that is highly relevant for dosing duration in inflammatory disease [100]. Thus, short-course vs. prolonged exposure may produce qualitatively different immune states, particularly in conditions where innate “memory-like” phenomena (tolerance/priming) shape pathology. Clinically, this argues for careful attention to (i) stage of disease (replicative vs. hyperinflammatory), (ii) kinetics of cytokine and IFN responses, and (iii) pharmacodynamic confirmation of target engagement within the relevant compartments (e.g., lung, monocytes/macrophages) [138,139,140,141].

The principal safety concern is that MEK inhibition could compromise protective immune functions—particularly when robust innate responses are needed for pathogen control or when immune competence is already reduced by comorbidities or concomitant therapies. However, risks are not limited to “too little immunity.” In some settings, MEK inhibition can exacerbate specific inflammatory outputs by removing negative regulatory constraints. A striking example is the report that MEK inhibitors increased mortality in an LPS-induced inflammation model through an IL-12–NO axis, illustrating that pathway perturbation can shift cytokine wiring toward lethal inflammatory effector mechanisms [113]. While LPS is not sterile inflammation, this model directly reflects TLR4-driven systemic inflammation, making it a relevant warning signal for any indication with endotoxin translocation, bacterial co-infection, or PRR-driven cytokine storm.

A second risk dimension is interferon pathway dysregulation, particularly in IFN-high autoimmune endotypes driven by endosomal TLR7/9 sensing [142]. MEK/ERK can act as a brake on IFN production in certain myeloid contexts, and therefore MEK blockade could, under specific cellular wiring, increase type I IFN outputs and potentially worsen IFN-driven autoimmunity [143,144]. This supports a biomarker-guided strategy in autoimmune diseases, distinguishing TNF/IL-1-dominant from IFN-dominant inflammatory programs before considering MEK inhibition.

MEK inhibitors are most likely to be deployed either (i) as short-course host-directed agents in acute infection, potentially combined with DAAs and standard anti-inflammatory care, or (ii) as adjunctive anti-inflammatory modulators in chronic inflammatory disease. In infection, combination therapy can be attractive because it pairs direct antiviral suppression with a host-targeted resistance barrier and immunomodulation. However, combination regimens increase complexity and raise the importance of mechanistic monitoring, because immunosuppressive or immunostimulatory effects may be synergistic (beneficial or harmful) depending on context [136,145,146]. In chronic inflammatory disease, combinations with cytokine-targeted biologics might lower required doses of each agent and reduce toxicity, but systematic evidence remains limited. Given the network role of MEK/ERK, rational combinations should be based on clearly defined immune endotypes and validated pharmacodynamic markers [132,147,148].

A pragmatic translational strategy for the clinical application of MEK inhibitors requires careful identification of disease settings in which modulation of TLR-driven inflammatory pathways is most likely to confer benefit while minimizing potential risks. Therapeutic prioritization should focus on conditions characterized by excessive TLR2- or TLR4-dependent cytokine responses, as well as acute viral infections in which phases of active viral replication overlap with hyperinflammatory immune activation and allow for short-course host-directed intervention [1,5,139]. In this context, stratification of patients according to inflammatory endotypes may be particularly important, as diseases dominated by TNF- or IL-1-driven pathways may respond differently to MEK inhibition than interferon-high conditions, where pharmacological blockade of the MEK/ERK axis has been shown to enhance type I interferon responses under specific circumstances [143,148].

Equally critical for successful translation is the optimization of dosing strategies that account for the dynamic nature of MEK/ERK signaling in innate immune cells. Short, well-timed treatment regimens aligned with disease kinetics may mitigate pathological inflammation while preserving essential host defense mechanisms, whereas prolonged pathway inhibition can alter feedback regulation and potentially reprogram inflammatory responses toward heightened cytokine production [14,136]. Integration of pharmacodynamic monitoring into clinical development programs therefore represents a key requirement, including assessment of pathway engagement biomarkers such as ERK phosphorylation in relevant immune compartments and downstream cytokine or interferon signatures that reflect the intended immunomodulatory effects [149,150]. In parallel, vigilance for mechanism-based safety signals remains essential, as experimental evidence indicates that MEK inhibition can in certain contexts lead to unexpected inflammatory rewiring, including enhanced IL-12–nitric oxide–mediated pathology and increased susceptibility to secondary infections in critically ill or co-infected patients [113].

Taken together, MEK inhibitors hold considerable promise as host-directed therapeutic agents at the interface of TLR-driven inflammation and infectious disease. However, their successful clinical application will depend on mechanistically informed patient selection, precise temporal control of treatment, and the integration of robust biomarker frameworks capable of distinguishing beneficial immunomodulation from detrimental immune dysregulation. Future progress in this field will require close alignment of clinical trial design with advances in systems-level understanding of innate immune signaling networks and feedback regulation, thereby enabling the identification of disease contexts in which MEK inhibition can reshape inflammatory trajectories toward improved clinical outcome.

## Figures and Tables

**Table 1:** Pharmacological inhibitors targeting the Tpl2–MEK–ERK signaling axis and their relevance for immune modulation.

Target Within Pathway	Representative Inhibitor(s)	Primary Mechanism of Action	Immune/Inflammatory Relevance	Translational Status	Key References
Tpl2 (MAP3K8/COT)	Experimental Tpl2 inhibitors	Blocks upstream activation of MEK1/2 downstream of TLR–MyD88 signaling	Reduces TLR-induced TNF-α, IL-6, and IL-1β; modulates inflammatory feedback	Preclinical/experimental	[45,55]
MEK1/2	PD98059, U0126	Prevents phosphorylation and activation of ERK1/2	Widely used in mechanistic in vitro studies of TLR signaling and cytokine regulation	Experimental tool compounds	[56,57,58,59,60]
MEK1/2	Selumetinib, Trametinib, Cobimetinib, Binimetinib	Highly selective MEK1/2 inhibition	Dual immunomodulatory effects in infection and inflammation; host-directed antiviral potential; modulation of cytokine release; dendritic cell activation; feedback signaling	Clinically approved in oncology and related indications (including Cobimetinib-based combination regimens)	[53,61,62,63]
MEK1/2	Zapnometinib	Selective MEK1/2 inhibition with host-directed antiviral activity	Suppresses virus-supportive host signaling pathways; modulates TLR-driven cytokine responses and excessive inflammation	Clinical development for viral infections/host-directed therapy	[64,65,66,67,68,69,70,71,72]
ERK1/2	SCH772984, Ulixertinib	Direct inhibition of ERK kinase activity	Used to distinguish MEK- from ERK-specific effects	Preclinical/early clinical	[73,74]
Negative feedback regulators	DUSP modulators (experimental)	Alters ERK-associated feedback termination	Affects signal duration and inflammatory tolerance	Experimental	[75]

Abbreviations: TLR, Toll-like receptor; Tpl2, tumor progression locus 2; MEK, mitogen-activated protein kinase kinase; ERK, extracellular signal-regulated kinase; DUSP, dual-specificity phosphatase.

**Table 2:** Major MEK inhibitors relevant to immune modulation. Summary of representative MEK inhibitors, their pharmacological characteristics, clinical status, and relevance for immune modulation.

Inhibitor	Clinical Status	Mechanism	Main Indications	Immunological Relevance
PD98059	Experimental	MEK1 inhibitor	Research tool	Early studies on TLR signaling
U0126	Experimental	MEK1/2 inhibitor	Research tool	Suppresses cytokine production
Selumetinib	Clinical	Allosteric MEK1/2 inhibitor	Cancer	Immune modulation
Trametinib	Approved	MEK1/2 inhibitor	Melanoma and other cancers	Antiviral and anti-inflammatory effects
Cobimetinib	Approved	MEK1/2 inhibitor	Melanoma	Alters dendritic cell and T-cell responses
Binimetinib	Approved	MEK1/2 inhibitor	Melanoma	Immunomodulatory effects
Zapnometinib (ATR-002)	Clinical development	Non-ATP-competitive MEK1/2 inhibitor	Viral infections	Host-directed antiviral and immune modulation

## Data Availability

No new data were created or analyzed in this study. Data sharing is not applicable to this article.

## Abbreviations

The following abbreviations are used in this manuscript:

DAA	Direct-Acting Antiviral
DAMP	Damage-Associated Molecular Pattern
DC	Dendritic Cell
DUSP	Dual Specificity Phosphatase
ERK	Extracellular Signal-Regulated Kinase
IFN	Interferon
IKK	IκB Kinase
IL	Interleukin
IRF	Interferon Regulatory Factor
JNK	c-Jun N-terminal Kinase
LPS	Lipopolysaccharide
MAPK	Mitogen-Activated Protein Kinase
MEK	MAPK/ERK Kinase (Mitogen-Activated Protein Kinase Kinase)
mRNA	Messenger ribonucleic acid
NF-κB	Nuclear Factor kappa B
NO	Nitric Oxide
PD	Pharmacodynamics
pDC	Plasmacytoid Dendritic Cell
PK	Pharmacokinetics
PRR	Pattern Recognition Receptor
RAF	Rapidly Accelerated Fibrosarcoma (RAF kinase)
Ras	Rat Sarcoma (small GTPase)
TAK	Transforming Growth Factor-β-Activated Kinase
TBK	TANK-Binding Kinase
TLR	Toll-Like Receptor
TNF	Tumor Necrosis Factor
Tpl2	Tumor Progression Locus 2
Treg	Regulatory T Cell
TRIF	TIR-domain-containing adapter-inducing interferon-β
